# A generalized solution procedure for in-plane free vibration of rectangular plates and annular sectorial plates

**DOI:** 10.1098/rsos.170484

**Published:** 2017-08-16

**Authors:** Siyuan Bao, Shuodao Wang

**Affiliations:** 1 School of Civil Engineering, Suzhou University of Science and Technology, Suzhou, Jiangsu 215011, China; 2 School of Mechanical and Aerospace Engineering, Oklahoma State University, Stillwater, OK 74078, USA

**Keywords:** rectangular plate, annular sectorial plate, in-plane vibration, improved Fourier–Ritz method, logarithmic radial variable

## Abstract

A generalized solution procedure is developed for in-plane free vibration of rectangular and annular sectorial plates with general boundary conditions. For the annular sectorial plate, the introduction of a logarithmic radial variable simplifies the basic theory and the expression of the total energy. The coordinates, geometric parameters and potential energy for the two different shapes are organized in a unified framework such that a generalized solving procedure becomes feasible. By using the improved Fourier–Ritz approach, the admissible functions are formulated in trigonometric form, which allows the explicit assembly of global mass and stiffness matrices for both rectangular and annular sectorial plates, thereby making the method computationally effective, especially when analysing annular sectorial plates. Moreover, the improved Fourier expansion eliminates the potential discontinuity of the original normal and tangential displacement functions and their derivatives in the entire domain, and accelerates the convergence. The generalized Fourier–Ritz approach for both shapes has the characteristics of generality, accuracy and efficiency. These features are demonstrated via a few numerical examples.

## 1. Introduction

The in-plane vibration of built-up structures is found to have a significant effect on the sound radiation and transmission of vibration energies [1,2]. In-plane vibration analysis is also important when inspecting the hulls of ships under the impacts of boundary flow, and similarly for studying the dynamic behaviours of composite shells of an aeroplane flying through turbulence [3]. Therefore, a better understanding in the in-plane vibration behaviours of plates is important in the design of similar structures. For the in-plane vibration of plate structures, several analytical solutions are developed, e.g. the variational method by Kantorovich–Krylov in [4], the superposition method by Gorman [5], the direct separation of variables and eigenvalue-problem approach by Xing and Liu [3,6], the strong form of the governing equation solved via a two-dimensional improved Fourier series by Du *et al*. [7] and the Ritz method based on a set of trigonometric functions by Dozio [8], just to name a few. Bardell *et al*. [9] presented, for the first time, the in-plane frequencies of rectangular plates under many combined boundary conditions. Some new methods have also been presented recently: for example the dynamic stiffness method by Nefovska-Danilovic & Petronijevic [10], and the solution for free vibration of thin rectangular plates with elastic boundary and internal line supports based on improved Fourier–Ritz method by Shi *et al.* [11]. For a circular disk, Onoe [12,13] presented an exact solution on the basis of Love's theory. Chen & Liu [14] proposed a general solution of the governing differential equations for thin plates in different shapes with boundary conditions satisfied in a least-square sense. Holland [15] as well as Farag & Pan [16] adopted the trigonometric and Bessel functions to study in-plane vibration of circular plates. A few different methods are also proposed: the transfer matrix method by Irie *et al*. [17], the generalized Rayleigh–Ritz method by Bashmal *et al*. [18], Hamilton's principle by Park [19], the variational approximation procedure by Seok & Tiersten [20] and the stress–strain–displacement expressions by Ravari & Forouzan [21]. Vladimir *et al*. [22] applied the potential theory to study the free in-plane vibration of rectangular, annular and circular plates. Kim *et al*. [23] discussed the in-plane vibration of a circular plate based on the assumption that the mode shapes are dependent on the number of nodal diameters. Singh & Muhammad [24] presented a numerical method to study the free in-plane vibration of the isotropic non-rectangular plate, in which the plate is meshed and the displacement field and the coordinate field are interpolated separately. Wang *et al*. [25] used a modified Fourier–Ritz approach [26,27] to solve the free in-plane vibration of orthotropic circular, annular and sectorial plates subjected to general boundary conditions. Recently, the modified Fourier series technique has been extended to study the in-plane vibration of plate and shell structures with general boundary conditions by the modified Ritz method [25,28–31].

However, in this large volume of literature, plates of different shapes are always treated separately and solved by different approaches. No generalized solution has been presented for both rectangular and circular shapes. In this work, the basic model for rectangular plates is briefly reviewed, then the basic theory for annular sectorial plates is modified by introducing a logarithmic radial variable, the theories are then formulated in a unified framework to account for both rectangular and annular sectorial plates. A few numerical examples are presented to demonstrate the versatility of the generalized approach.

## 2. Theoretical formulations

### 2.1. Orthotropic rectangular plates

The Rayleigh–Ritz method combined with the artificial spring technique [11] is briefly introduced here for analysing in-plane vibration problems of orthotropic rectangular plates.

**Figure 1.**
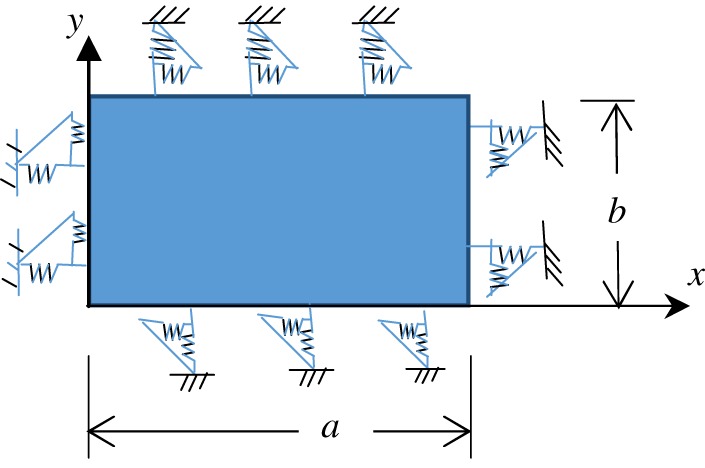
An orthotropic rectangular plate with arbitrary in-plane elastic supports.

Consider an orthotropic plate with length *a* and width *b* as shown in figure 1. On all the sides of the plate two groups of boundary elastic springs are arranged along the normal and tangential directions, to simulate the boundary conditions. By assigning the stiffness of the boundary springs with various values, we can impose different boundary conditions on the mid-surface of the plate edges. For the orthotropic rectangular plate, based on the strain–stress relationship, the boundary conditions can be expressed as

2.1 $$k_{xi}^{U}u={\left(-1\right)}^{i}\;\left(A_{xx}\frac{\partial u}{\partial x}+A_{xy}\frac{\partial v}{\partial y}\right),\;k_{xi}^{V}v={\left(-1\right)}^{i}G_{xy}\;\left(\frac{\partial u}{\partial y}+\frac{\partial v}{\partial x}\right)\;(i=0,1)$$

and

2.2 $$k_{yi}^{V}u={\left(-1\right)}^{i}\;\left(A_{yx}\frac{\partial u}{\partial x}+A_{yy}\frac{\partial v}{\partial y}\right),\;k_{yi}^{U}v={\left(-1\right)}^{i}G_{xy}\;\left(\frac{\partial u}{\partial y}+\frac{\partial v}{\partial x}\right)\;(i=0,1),$$

where $A_{xx}=E_{x}/1-\mu_{x}\mu_{y},\;A_{yy}=E_{y}/1-\mu_{x}\mu_{y},A_{xy}=A_{yx}=\mu_{x}E_{y}/1-\mu_{x}\mu_{y}$ are the in-plane stretch stiffness, *G_xy_* is the shear Young's modulus, *E_z_* and *μ_z_* (*z* = *x* or *y*) are Young's moduli and Poisson's ratio in the *x* and *y* directions, respectively. The parameter $k_{\gamma }^{\delta }$ stands for the attached spring stiffness, with its superscript *δ *= *U*,*V* indicating the *x* and *y* directions and the subscript *γ* = *x*0, *y*0, *x*1, *y*1 referring to the left, bottom, right, and top edges of the corresponding spring, respectively. For example, $k_{x0}^{U}$ denotes the spring stiffness in the *x* direction along the edge at *x* = 0. A clamped boundary can be readily obtained by setting the spring coefficients to infinity for both the normal and tangential restraining springs. The total potential energy of the plate, consisting of the strain energy of the plate and the potential energy stored in the boundary springs, can be expressed as:

2.3 $$\begin{array}{rl} V & =\frac{h}{2}\int_{0}^{a}\int_{0}^{b}\left[A_{xx}{\left(\frac{\partial u}{\partial x}\right)}^{2}+A_{yy}{\left(\frac{\partial v}{\partial y}\right)}^{2}+2A_{xy}\frac{\partial u}{\partial x}\left(\frac{\partial v}{\partial y}\right)+G_{xy}{\left(\frac{\partial u}{\partial y}+\frac{\partial v}{\partial x}\right)}^{2}\right]\;\text{d}x\text{d}y\\ & \;+\frac{1}{2}\int_{0}^{b}\left[{\left(k_{x0}^{U}u^{2}+k_{x1}^{V}v_{}^{2}\right)|}_{x=0}+{\left(k_{x1}^{U}u^{2}+k_{x1}^{V}v_{}^{2}\right)|}_{x=a}\right]\;\text{d}y\\ & \;+\frac{1}{2}\int_{0}^{a}\left[{\left(k_{y0}^{U}u^{2}+k_{y0}^{V}v_{}^{2}\right)|}_{y=0}+{\left(k_{yb}^{U}u^{2}+k_{yb}^{V}v_{}^{2}\right)|}_{y=b}\right]\;\text{d}x. \end{array}$$

The kinetic energy *T* of the plate is expressed as:

2.4 $$T=\frac{\rho h}{2}\int_{0}^{a}\int_{0}^{b}\left[{\left(\frac{\partial u}{\partial t}\right)}^{2}+{\left(\frac{\partial v}{\partial t}\right)}^{2}\right]\;\text{d}x\;\text{d}y.$$

where *ρ* is the plate mass density. Considering an harmonic motion with frequency *ω*, i.e.

2.5 $$\begin{array}{rl} u(x,y,t) & =u(x,y){\text{e}}^{j\omega t}=u{\text{e}}^{j\omega t}\\ \;\text{and}\;v(x,y,t) & =v(x,y){\text{e}}^{j\omega t}=v{\text{e}}^{j\omega t}, \end{array}\}$$

the maximum strain energy *V*_max_ and the maximum kinetic energy *T*_max_ for the plate are

2.6 $$\begin{array}{rl} V_{\max} & =\frac{h}{2}\int_{0}^{a}\int_{0}^{b}\left[A_{xx}{\left(\frac{\partial u}{\partial x}\right)}^{2}+A_{yy}{\left(\frac{\partial v}{\partial y}\right)}^{2}+2A_{xy}\frac{\partial u}{\partial x}\left(\frac{\partial v}{\partial y}\right)+G_{xy}{\left(\frac{\partial u}{\partial y}+\frac{\partial v}{\partial x}\right)}^{2}\right]\;\text{d}x\text{d}y\\ & \;+\frac{1}{2}\int_{0}^{a}\left[{\left(k_{x0}^{U}u^{2}+k_{x1}^{V}v_{}^{2}\right)|}_{x=0}+{\left(k_{xa}^{U}u^{2}+k_{xa}^{V}v_{}^{2}\right)|}_{x=a}\right]\;\text{d}y\\ & \;+\frac{1}{2}\int_{0}^{b}\left[{\left(k_{y0}^{U}u^{2}+k_{y0}^{V}v_{}^{2}\right)|}_{\theta =0}+{\left(k_{yb}^{U}u^{2}+k_{yb}^{V}v_{}^{2}\right)|}_{y=b}\right]\;\text{d}x \end{array}$$

2.7 $$\begin{array}{rl} \;\text{and}\;T_{\max} & =\frac{\rho h\omega^{2}}{2}\int_{0}^{a}\int_{0}^{b}\left(u^{2}+v^{2}\right)\;\text{d}x\text{d}y, \end{array}$$

respectively. By using the Ritz method, the energy function is defined by Lagrangian function as

2.8 L=V−T.

### 2.2. Orthotropic annular sectorial plates

**Figure 2.**
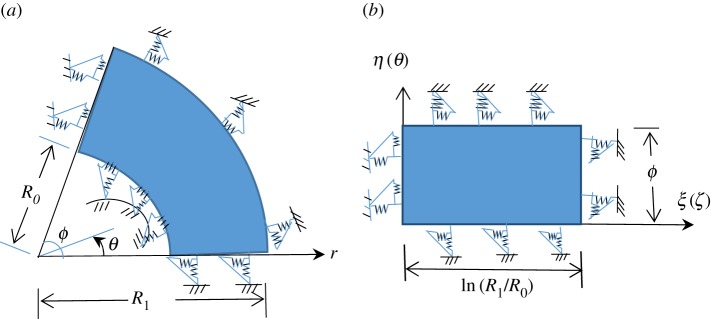
Schematics of (*a*) an orthotropic annular sectorial plate and (*b*) the corresponding generalized model.

Consider an orthotropic annular sectorial plate with uniform thickness *h*, inner radius *R*_0_, outer radius *R*_1_, and sector angle *φ* as shown figure 2*a*. For general supported orthotropic annular sectorial plates, based on the force equilibrium relationship at the four edges, the boundary conditions corresponding to the elastic spring can be expressed as:

2.9 $$k_{Ri}^{U}u=A_{rr}\frac{\partial u}{\partial r}+A_{r\theta }\frac{1}{r}\left(u+\frac{\partial v}{\partial \theta }\right),\;k_{Ri}^{V}v=G_{r\theta }\left(\frac{1}{r}\frac{\partial u}{\partial \theta }+\frac{\partial v}{\partial r}-\frac{v}{r}\right)\;(i=0,1)$$

and

2.10 $$k_{ti}^{V}u=A_{\theta r}\frac{\partial u}{\partial r}+A_{\theta \theta }\frac{1}{r}\left(u+\frac{\partial v}{\partial \theta }\right),\;k_{ti}^{U}v=-G_{r\theta }\left(\frac{1}{r}\frac{\partial u}{\partial \theta }+\frac{\partial v}{\partial r}-\frac{v}{r}\right)\;(i=0,1),$$

where $A_{rr}=E_{r}/1-\mu_{r}\mu_{\theta },\;A_{\theta \theta }=E_{\theta }/1-\mu_{r}\mu_{\theta },\;A_{r\theta }=\mu_{r}E_{\theta }/1-\mu_{r}\mu_{\theta }$ are the in-plane stretch stiffness, *G_rθ_* is the shear Young's modulus, *E_z_* and *μ_z_* (*z* = *r* or *θ*) are Young's moduli and Poisson's ration in the *r* and *θ* directions of the orthotropic plate, respectively.

Arbitrary elastic support conditions are imposed by assigning massless normal and tangential springs along each edge. Similar to the case of a rectangular plate, the symbol $k_{\gamma }^{\delta }$ denotes the spring restraining stiffness value, with *δ* =* U*, *V* denoting the *r* and *θ* directions and *γ *= *R*_0_, *θ*_0_, *R*_1_, *θ*_1_ referring to the inner, bottom, outer, and upper edges, respectively.

The strain energy *V_pl_* of the sectorial plate is written as

2.11 $$V_{pl}=\frac{h}{2}\int_{R_{0}}^{R_{1}}\int_{0}^{\phi }\left[A_{rr}{\left(\frac{\partial u}{\partial r}\right)}^{2}+\frac{A_{\theta \theta }}{r^{2}}{\left(u+\frac{\partial v}{\partial \theta }\right)}^{2}+2\frac{A_{r\theta }}{r}\frac{\partial u}{\partial r}\left(\frac{\partial v}{\partial \theta }+u\right)+G_{r\theta }{\left(\frac{1}{r}\frac{\partial u}{\partial \theta }+\frac{\partial v}{\partial r}-\frac{v}{r}\right)}^{2}\right]\;r\text{d}r\text{d}\theta .$$

The potential energy *V_s_* stored in the boundary springs is obtained as

2.12 $$\begin{array}{rl} V_{s} & =\frac{1}{2}\int_{0}^{\phi }[R_{0}{\left(k_{r0}^{U}u^{2}+k_{r0}^{V}v_{}^{2}\right)|}_{\rho =\ln a}+{R_{1}(k_{r1}^{U}u^{2}+k_{r1}^{V}v_{}^{2})|}_{\rho =\ln b}]\;\text{d}\theta \\ & \;+\frac{1}{2}\int_{R_{0}}^{R_{1}}\left[{\left(k_{\theta 0}^{U}u^{2}+k_{\theta 0}^{V}v_{}^{2}\right)|}_{\theta =0}+{\left(k_{\theta 1}^{U}u^{2}+k_{\theta 1}^{V}v_{}^{2}\right)|}_{\theta =\phi }\right]\;r\text{d}r. \end{array}$$

The total potential energy is

2.13 $$\begin{array}{rl} V & =\frac{h}{2}\int_{R_{0}}^{R_{1}}\int_{0}^{\phi }\left[A_{rr}{\left(\frac{\partial u}{\partial r}\right)}^{2}+A_{\theta \theta }\frac{1}{r^{2}}{\left(u+\frac{\partial v}{\partial \theta }\right)}^{2}+2A_{r\theta }\frac{1}{r}\frac{\partial u}{\partial r}\left(\frac{\partial v}{\partial \theta }+u\right)+G_{r\theta }{\left(\frac{1}{r}\frac{\partial u}{\partial \theta }+\frac{\partial v}{\partial r}-\frac{v}{r}\right)}^{2}\right]\;r\text{d}r\text{d}\theta \\ & \;+\frac{1}{2}\int_{0}^{\phi }\left[R_{0}{\left(k_{r0}^{U}u^{2}+k_{r0}^{V}v_{}^{2}\right)|}_{r=R_{0}}+{R_{1}(k_{r1}^{U}u^{2}+k_{r1}^{V}v_{}^{2})|}_{r=R_{1}}\right]\;\text{d}\theta \\ & \;+\frac{1}{2}\int_{R_{0}}^{R_{1}}\left[{\left(k_{\theta 0}^{U}u^{2}+k_{\theta 0}^{V}v_{}^{2}\right)|}_{\theta =0}+{\left(k_{\theta 1}^{U}u^{2}+k_{\theta 1}^{V}v_{}^{2}\right)|}_{\theta =\phi }\right]\;\text{d}r. \end{array}$$

The kinetic energy *T* is expressed as:

2.14 $$T=\frac{\rho h}{2}\int_{R_{1}}^{R_{1}}\int_{0}^{\phi }\left[{\left(\frac{\partial u}{\partial t}\right)}^{2}+{\left(\frac{\partial v}{\partial t}\right)}^{2}\right]\;r\text{d}r\text{d}\theta ,$$

where *ρ* is the plate mass density. Considering an harmonic motion with frequency *ω*, i.e.

2.15 $$\begin{array}{rl} u(r,\theta ,t) & =\bar{u}(r,\theta ){\text{e}}^{j\omega t}=\bar{u}{\text{e}}^{j\omega t}\\ \;\text{and}\;v(r,\theta ,t) & =\bar{v}(r,\theta ){\text{e}}^{j\omega t}=\bar{v}{\text{e}}^{j\omega t}, \end{array}\}$$

the maximum strain energy *V*_max_ and the maximum kinetic energy *T*_max_ for the plate are

2.16 $$\begin{array}{rl} V_{\max} & =\frac{h}{2}\int_{R_{0}}^{R_{1}}\int_{0}^{\phi }\left[A_{rr}{\left(\frac{\partial \bar{u}}{\partial r}\right)}^{2}+\frac{A_{\theta \theta }}{r^{2}}{\left(\bar{u}+\frac{\partial \bar{v}}{\partial \theta }\right)}^{2}+2\frac{A_{r\theta }}{r}\frac{\partial \bar{u}}{\partial r}\left(\frac{\partial \bar{v}}{\partial \theta }+\bar{u}\right)+G_{r\theta }{\left(\frac{1}{r}\frac{\partial \bar{u}}{\partial \theta }+\frac{\partial \bar{v}}{\partial r}-\frac{\bar{v}}{r}\right)}^{2}\right]r\text{d}r\text{d}\theta \\ & \;+\frac{1}{2}\int_{0}^{\phi }\left[R_{0}{\left(k_{R0}^{U}{\bar{u}}^{2}+k_{R0}^{V}{\bar{v}}_{}^{2}\right)|}_{r=R_{0}}+{R_{1}(k_{R1}^{U}{\bar{u}}^{2}+k_{R1}^{V}{\bar{v}}_{}^{2})|}_{r=R_{1}}\right]\;\text{d}\theta \\ & \;+\frac{1}{2}\int_{R_{0}}^{R_{1}}\left[{\left(k_{\theta 0}^{U}{\bar{u}}^{2}+k_{\theta 0}^{V}{\bar{v}}_{}^{2}\right)|}_{\theta =0}+{\left(k_{\theta 1}^{U}u^{2}+k_{\theta 1}^{V}{\bar{v}}_{}^{2}\right)|}_{\theta =\phi }\right]\;\text{d}r \end{array}$$

and

2.17 $$T_{\max}=\frac{\rho h\omega^{2}}{2}\int_{R_{0}}^{R_{1}}\int_{0}^{\phi }\left({\bar{u}}^{2}+{\bar{v}}^{2}\right)\;r\text{d}r\text{d}\theta ,$$

respectively.

To simplify the expressions in equation (2.16), a logarithmic radial variable is introduced based on the work of Yao *et al.* [32]

2.18 $$\varsigma =\ln\left(\frac{r}{R_{0}}\right).$$

Figure 2*b* shows the illustration of the plate using the logarithmic radial variable. The following relationships can be obtained

2.19 $$\begin{array}{rl} \text{d}r & =r\text{d}\varsigma \\ \;\text{and}\;\frac{\partial S(r,\theta )}{\partial r} & =\frac{1}{r}\frac{\partial S_{1}(\varsigma ,\theta )}{\partial \varsigma }, \end{array}\}$$

where *S*(*r*,*θ*) stands for an arbitrary function varying with *r* and *θ*, and *S*_1_(*ς*,*θ*) is the function *S*(*r*,*θ*) written in terms of *ζ* and *θ*. Substituting equation (2.19) into equations (2.13) and (2.14), we have

2.20 $$\begin{array}{rl} V_{\max} & =\frac{h}{2}\int_{0}^{L_{R}}\int_{0}^{\phi }\left[A_{rr}{\left(\frac{\partial u}{\partial \varsigma }\right)}^{2}+A_{\theta \theta }{\left(u+\frac{\partial v}{\partial \theta }\right)}^{2}+2A_{r\theta }\frac{\partial u}{\partial \varsigma }\left(\frac{\partial v}{\partial \theta }+u\right)+G_{r\theta }{\left(\frac{\partial u}{\partial \theta }+\frac{\partial v}{\partial \varsigma }-v\right)}^{2}\right]\;\text{d}\varsigma \text{d}\theta \\ & \;+\frac{1}{2}\int_{0}^{\phi }\left[R_{0}{\left(k_{R0}^{U}u^{2}+k_{R0}^{V}v_{}^{2}\right)|}_{\varsigma =0}+{R_{1}(k_{R1}^{U}u^{2}+k_{R1}^{V}v_{}^{2})|}_{\varsigma =L_{R}}\right]\;\text{d}\theta \\ & \;+\frac{1}{2}R_{0}\int_{0}^{L_{R}}\left[{\left(k_{\theta 0}^{U}u^{2}+k_{\theta 0}^{V}v_{}^{2}\right)|}_{\theta =0}+{\left(k_{\theta 1}^{U}u^{2}+k_{\theta 1}^{V}v_{}^{2}\right)|}_{\theta =\phi }\right]\;{\text{e}}^{\varsigma }\text{d}\varsigma \end{array}$$

and

2.21 $$T_{\max}=\frac{\rho h\omega^{2}}{2}R_{0}^{2}\int_{0}^{L_{R}}\int_{0}^{\phi }\left(u^{2}+v^{2}\right){\text{e}}^{2\varsigma }\text{d}\varsigma \text{d}\theta ,$$

respectively, where *L_R_* = ln(*R*_1_/*R*_0_).

### 2.3 Generalized theories for rectangular and annular sectorial plates

Similarities between the theories for rectangular and annular sectorial plates are summarized in this section. First, we formulate the unified expressions for the potential energy and the kinematic energy as

2.22 $$\begin{array}{rl} V_{\max} & =\frac{h}{2}\int_{0}^{\xi_{\max}}\int_{0}^{\eta_{\max}}\left[A_{11}{\left(\frac{\partial u}{\partial \xi }\right)}^{2}+A_{22}{\left(pu+\frac{\partial v}{\partial \eta }\right)}^{2}+2A_{12}\frac{\partial u}{\partial \xi }\left(\frac{\partial v}{\partial \eta }+pu\right)+G_{12}{\left(\frac{\partial u}{\partial \eta }+\frac{\partial v}{\partial \xi }-pv\right)}^{2}\right]\;\text{d}\xi \text{d}\eta \\ & \;+\frac{1}{2}\int_{0}^{\eta_{\max}}\left[{\text{e}}^{p\ln R_{0}}{\left(k_{R0}^{U}u^{2}+k_{R0}^{V}v_{}^{2}\right)|}_{\xi =0}+{{\text{e}}^{p\ln R_{1}}(k_{R1}^{U}u^{2}+k_{R1}^{V}v_{}^{2})|}_{\xi =\xi_{\max}}\right]\;\text{d}\eta \\ & \;+\frac{1}{2}R_{0}\int_{0}^{\xi_{\max}}\left[{\left(k_{\theta 0}^{U}u^{2}+k_{\theta 0}^{V}v_{}^{2}\right)|}_{\theta =0}+{\left(k_{\theta 1}^{U}u^{2}+k_{\theta 1}^{V}v_{}^{2}\right)|}_{\eta =\eta_{\max}}\right]\;{\text{e}}^{p\xi }\text{d}\xi \end{array}$$

and

2.23 $$T_{\max}=\frac{\rho h\omega^{2}}{2}R_{0}^{2p}\int_{0}^{\xi_{\max}}\int_{0}^{\eta_{\max}}\left(u^{2}+v^{2}\right){\text{e}}^{2p\xi }\text{d}\xi \text{d}\eta ,$$

respectively, where the shape parameter *p* is defined as

2.24 $$\begin{array}{ll} p=0 & \left(\text{for rectanglar plate}\right)\\ p=1 & (\text{for annular sectorial plate}). \end{array}\}$$

The material parameters are written in generalized forms as *A*_11_, *A*_12_, *A*_22_, *G*_12_, *E*_1_, *E*_2_, *μ*_1_, *μ*_2_ and the maximum lengths in *ξ* and *η* directions are *ξ*_max_ and *η*_max_, respectively. Table 1 shows the corresponding variables for rectangular and annular sectorial shapes, respectively.

**Table 1.** Variables and parameters in the generalized model.

generalized model	rectangular plate	annular sectorial plate
ξ	0 *≤ x ≤ a*	0 ≤* ζ *≤* *ln(*R*_1_/*R*_0_)
η	0 ≤ *y ≤ b*	0 ≤ *θ* ≤ *ϕ*
ρ	0	1
*A*_11_, *A*_12_ and *A*_22_	*A_xx_*, *A_xy_* and *A_yy_*	*A_rr_*, *A_rθ_* and *A_θθ_*
*G*_12_	*G_xy_*	*G_rθ_*
*E*_1_, *E*_1_	*E_x_*, *E_y_*	*E_r_*, *E_θ_*
*μ*_1_*, μ*_2_	*μ_x_, μ_y_*	*μ_r_, μ_θ_*
normalized frequency parameter	$\Omega =\omega b/\pi \sqrt{\rho /G_{xy}}$	$\Omega =2\omega R_{1}/\pi \sqrt{\rho /E_{r}(1-\mu_{r}\mu_{\theta })}$
normalized spring stiffness	$\tilde{K}=ka((1-\mu_{x}\mu_{y}\text{) /}E_{x})$	$\tilde{K}=kR_{0}((1-\mu_{r}\mu_{\theta }\text{) /}E_{r})$
spring stiffness	$0\leq \Gamma_{U(V)}\leq 10^{4}$	$0\leq \Gamma_{U(V)}\leq 10^{7}$

The displacements can be written as

2.25 $$\begin{array}{rl} u(\xi ,\eta ) & =\sum_{m=1}^{M}\sum_{n=1}^{N}a_{mn}f_{m}(\xi )g_{n}(\eta )\\ \;\text{and}\;v(\xi ,\eta ) & =\sum_{m=1}^{M}\sum_{n=1}^{N}b_{mn}f_{m}(\xi )g_{n}(\eta ), \end{array}\}$$

where *a_mn_* and *b_mn_* are unknown coefficients, *f_m_*(*ξ*) and *g_n_*(*θ*) are appropriate admissible functions, and *M* and *N* are the numbers of truncated terms in the series expansion. The proposed solution can be of arbitrary precision depending on the number of terms used in the series. In this work, the simple trigonometric series for constructing the two-direction displacements are selected as

2.26 $$\begin{array}{rl} f_{m}(\xi ) & =\left\{ \begin{array}{l} \sin\left(\xi \frac{m\pi }{\xi_{\max}}\right),m=1,2\\ \cos\left(\xi \frac{(m-3)\pi }{\xi_{\max}}\right),m\geq 3 \end{array}\right.\\ \;\text{and}\;g_{n}(\eta ) & =\left\{ \begin{array}{l} \sin\left(\eta \frac{n\pi }{\eta_{\max}}\right),n=1,2\\ \cos\left(\eta \frac{(n-3)\pi }{\eta_{\max}}\right),n\geq 3. \end{array}\right. \end{array}\}$$

Substitution of equation (2.25) into equations (2.22) and (2.23), and minimizing the energy function *L* = *U*_max_ − *T*_max_ with respect to the unknown coefficients *c_mn_* and *d_mn_* yield the following eigenvalue equations

2.27 $$\begin{array}{rl} & \sum_{r=1}^{M}\sum_{s=1}^{N}[K_{mnrs}^{uu}a_{rs}+K_{mnrs}^{uv}b_{rs}-\omega^{2}M_{mnrs}a_{rs}]=0\\ \;\text{and}\; & \sum_{r=1}^{M}\sum_{s=1}^{N}[K_{mnrs}^{vu}a_{rs}+K_{mnrs}^{vv}b_{rs}-\omega^{2}M_{mnrs}b_{rs}]=0, \end{array}\}$$

where the elements of the global stiffness matrix $K_{mnrs}^{uu},K_{mnrs}^{uv},K_{mnrs}^{vu},K_{mnrs}^{vv}$ are given by

2.28 $$\begin{array}{rl} K_{mnrs}^{uu} & =A_{11}I_{mr}^{11}I_{ns}^{00}+G_{12}I_{mr}^{00}I_{ns}^{11}+pA_{22}I_{mr}^{00}I_{ns}^{00}+pA_{12}I_{ns}^{00}(I_{mr}^{10}+I_{mr}^{01})\\ & \;+{\text{e}}^{p\ln R_{0}}(E_{mr}^{0}J_{ns}^{r0U}+E_{mr}^{1}J_{ns}^{r1U})+({\text{e}}^{p\ln R_{0}}J_{mr}^{t0U}E_{ns}^{0}+{\text{e}}^{p\ln R_{1}}J_{mr}^{t1U}E_{ns}^{1})\\ K_{mnrs}^{uv} & =pA_{22}I_{mr}^{00}I_{ns}^{01}+A_{12}I_{mr}^{10}I_{ns}^{01}+G_{12}(I_{mr}^{01}-pI_{mr}^{00})I_{ns}^{10}\\ \;\text{and}\;K_{mnrs}^{vv} & =A_{22}I_{mr}^{00}I_{ns}^{11}+G_{12}(pI_{mr}^{00}-pI_{mr}^{10}-pI_{mr}^{01}+I_{mr}^{11})I_{ns}^{00}\\ & \;+{\text{e}}^{p\ln R_{0}}(E_{mr}^{0}J_{ns}^{r0V}+E_{mr}^{1}J_{ns}^{r1V})+({\text{e}}^{p\ln R_{0}}J_{mr}^{t0V}E_{ns}^{0}+{\text{e}}^{p\ln R_{1}}J_{mr}^{t1V}E_{ns}^{1}), \end{array}\}$$

where *p*^2^ is replaced by *p* because *p* = *p*^2^, and the following quantities are defined:

2.29 $$\begin{array}{rl} E_{mr}^{0} & =f_{m}(0)f_{r}(0),E_{ns}^{0}=g_{n}(0)g_{s}(0)\\ \;\text{and}\;E_{mr}^{1} & =f_{m}(\xi_{\max})f_{r}(\xi_{\max}),E_{ns}^{1}=g_{n}(\eta_{\max})g_{s}(\eta_{\max}), \end{array}\}$$

2.30 $$\begin{array}{rl} I_{mr}^{\alpha \beta } & =\int_{0}^{\xi_{\max}}\frac{d^{\alpha }f_{m}}{d\xi^{\alpha }}\frac{d^{\beta }f_{r}}{d\xi^{\beta }}\text{d}\xi ,I_{ns}^{\alpha \beta }=\int_{0}^{\eta_{\max}}\frac{d^{\alpha }g_{n}}{d\eta^{\alpha }}\frac{d^{\beta }g_{s}}{d\eta^{\beta }}\text{d}\eta \\ \;\text{and}\;J_{mr}^{\gamma \delta } & =\int_{0}^{\xi_{\max}}k_{\gamma }^{\delta }f_{m}(\xi )f_{r}(\xi ){\text{e}}^{p\xi }\text{d}\xi ,J_{ns}^{\gamma \delta }=\int_{0}^{\phi }k_{\gamma }^{\delta }g_{n}(\eta )g_{s}(\eta )\text{d}\eta \end{array}\}$$

2.31 $$\;\text{and}\;P_{mr}=\int_{0}^{\xi_{\max}}f_{m}f_{r}{\text{e}}^{2p\xi }\text{d}\xi .$$

The elements of the global mass matrix $M_{mnrs}^{uu}$, $M_{mnrs}^{vv}$ are

2.32 $$\begin{array}{rl} M_{mnrs}^{uu} & =\rho h{\text{e}}^{2p\ln R_{0}}P_{mr}I_{ns}^{00}\\ \;\text{and}\;M_{mnrs}^{vv} & =M_{mnrs}^{uu}. \end{array}\}$$

When the spring stiffness of the elastic boundary is uniform along all the boundary edges, it can be found that

2.33 $$J_{mr}^{\gamma \delta }=k_{\gamma }^{\delta }I_{mr}^{00},J_{ns}^{\gamma \delta }=k_{\gamma }^{\delta }I_{ns}^{00}.$$

Equation (2.27) can also be written in the matrix form:

2.34 $$\left\{\left[\begin{array}{cc} K^{uu} & K^{uv}\\ K^{vu} & K^{vv} \end{array}\right]-\omega^{2}\left[\begin{array}{cc} M_{}^{uu} & 0\\ 0 & M_{}^{vv} \end{array}\right]\right\}\;\left\{\begin{array}{c} A^{u}\\ B^{v} \end{array}\right\}=0,$$

where

2.35 $$\begin{array}{rl} A^{u} & =\{a_{11},a_{12},c_{13},\ldots ,a_{1N},a_{21},\ldots ,a_{rs},\ldots ,a_{MN}\}\\ \;\text{and}\;B^{v} & =\{b_{11},b_{12},b_{13},\ldots ,b_{1N},b_{21},\ldots ,b_{rs},\ldots ,b_{MN}\}. \end{array}\}$$

Equation (2.34) corresponds to an eigenvalue problem, whose eigenvalues correspond to the frequencies of the in-plane free vibration of the plates. Substitution of the obtained eigenvectors into equation (2.25) yields the corresponding mode shapes.

In the present method, the analytic form of the integrals involved in the mass and stiffness matrix can be obtained. For the case of uniform spring stiffness, when equation (2.26) is selected as admissible functions, the formulations of all the sub-matrices in equation (2.34), such as ***K***^*uu*^, ***K***^*vv*^, ***K***^*uv*^, ***M***^*uu*^, ***M***^*vv*^, can all be obtained explicitly with the aid of the basic integration formulae in the electronic supplementary material. Further, according to the form of the admissible functions selected, these matrixes can also be assembled by four block sub-matrices as

2.36 $$Z=\left[\begin{array}{cc} Z_{11} & Z_{12}\\ Z_{21} & Z_{22} \end{array}\right],$$

where ***Z*** stands for ***K***^*uu*^, ***K***^*vv*^, ***K***^*uv*^, ***M***^*uu*^ or ***M***^*vv*^, and ***Z***_11_, ***Z***_12,_ ***Z***_21_, ***Z***_22,_ are 2 × 2, 2 × (*MN* − 2), (*MN* − 2) × 2, (*MN* − 2) × (*MN* − 2) matrices, respectively. If the chosen admissible functions in equation (2.26) do not include the first two items, this corresponds to the case of normal Fourier expansion for the in-plane displacements, and the matrix ***Z*** degenerates to ***Z***_22_ in equation (2.36).

When calculating the sub matrix **Z***_ij_*(*i,j* = 1,2) for the improved Fourier series, the base functions $f_{m}(\xi ),f_{r}(\xi ),g_{n}(\eta )$and *g_s_*(*η*) are all the trigonometric functions, and therefore the orthogonal characteristics of the Fourier series can be exploited

2.37 $$\begin{array}{rl} \int_{0}^{L}\cos(\lambda_{m}x)\cos(\lambda_{n}x)\text{d}x & =0,m\neq n,\\ \int_{0}^{L}\sin(\lambda_{m}x)\sin(\lambda_{n}x)\text{d}x & =0,m\neq n\\ \;\text{and}\;\int_{0}^{L}\sin(\lambda_{m}x)\cos(\lambda_{n}x)\text{d}x & =0,m=n, \end{array}\}$$

where λ*_m _*= *mπ*/*L*, λ*_n _*= *nπ*/*L*. This orthogonal property is useful in obtaining the elements of matrix ***Z*** and many of which are found to be zero.

## 3. Numerical examples and discussions

Four different materials are used in the examples, and their properties are listed in table 2. The shear elasticity *G*_12_ is defined as

3.1 $$\begin{array}{rl} G_{xy} & =\frac{E_{y}/2}{\left(1-\mu_{x}\mu_{y}\right)}\;(\text{for rectangular plate})\\ G_{r\theta } & =\frac{A_{rr}/2}{\left(1-\mu_{r}\mu_{\theta }\right)}\;(\text{for annular sectorial plate}). \end{array}\}$$

**Table 2.** Material properties used in this study.

shape	materials	*E_ξ_*/*E_η_*	*E_η_* (GPa)	*μ_ξ_*	*ρ*(kg/m^3^)
rectangular	*A*	*E_x_ /E_y_* = 2	*E_y_* = 70	*μ_x_* = 0.3	7850
annular sectorial	*B*	*E_r_ /E_θ_* = 40	*E_θ_* = 70	*μ_r_* = 0.3	7850
	*C*	*E_r_ /E_θ_* = 20	*E_θ_* = 70	*μ_r_* = 0.3	7850
	*D*	*E_r_ /E_θ_* = 1	*E_θ_* = 70	*μ_r_* = 0.3	7850

The boundary conditions of rectangular and annular sectorial plates are denoted by a four-letter symbol, with each letter standing for the boundary condition of one edge starting from the left edge at *ξ *=* *0. For example, for a rectangular plate, the symbol E^1^CFS^1^ denotes E^1^ type elastic boundary condition at *x *=* *0, clamped boundary condition at *y *=* *0, free boundary condition at *x *=* a*, and S^1^ type simply supported at *y = b*. For an annular sectorial plate, the same symbol denotes the corresponding boundary conditions at *r *=* R*_0_, *θ *=* *0, *r = R*_1_, *θ *=* ϕ*, respectively. The stiffness values of the boundary spring are listed in table 3 for the different types of boundary conditions. 10^4^ and 10^7^ are shown to be appropriate values for the non-dimensional spring stiffness for clamped boundary condition in rectangular and annular sectorial plates, respectively [11].

**Table 3.** Non-dimensional spring stiffness values for general boundary conditions.

		at *ξ *= constant			at *η *= constant		
shape	boundary condition	essential conditions	*Γ_U_*	*Γ_V_*	essential conditions	*Γ_U_*	*Γ_V_*
rectangular	free (F)	*σ_x_* = 0, *τ_xy_* = 0	0	0	*σ_y_* = 0, *τ_xy_* = 0	0	0
	clamped (C)	*u* = 0, *v* = 0	10^4^	10^4^	*u* = 0, *v* = 0	10^4^	10^4^
	simple-support (S^1^)	*v* = 0, *σ_x_* = 0	0	10^4^	*u* = 0, *σ_y_* = 0	10^4^	0
	simple-support (S^2^)	*u* = 0, *τ_xy_* = 0	10^4^	0	*u* = 0, *σ_y_* = 0	10^4^	0
	elastic 1 (E^1^)	*u* ≠ 0, *τ_xy_* = 0	10	0	*u* ≠ 0, *τ_xy_* = 0	10	0
	elastic 2 (E^2^)	*v* ≠ 0, *σ_x_* = 0	0	10^2^	*v* ≠ 0, *σ_y_* = 0	0	10^2^
	elastic 3 (E^3^)	*u* ≠ 0, *v* ≠ 0	10^3^	10^3^	*u* ≠ 0, *v* ≠ 0	10^3^	10^3^
annular sectorial	free (F)	*σ_r_* = 0, *τ_rθ_* = 0	0	0	*σ_r_* = 0, *τ_rθ_* = 0	0	0
	clamped (C)	*u* = 0, *v* = 0	10^7^	10^7^	*u* = 0, *v* = 0	10^7^	10^7^
	simple-support (S^1^)	*u* = 0, *σ_r_* = 0	0	10^7^	*u* = 0, *σ_θ_* = 0	10^7^	0
	simple-support (S^2^)	*u* = 0, *τ_rθ_* = 0	10^7^	0	*u* = 0, *σ_θ_* = 0	10^7^	0
	elastic 1 (E^1^)	*u* ≠ 0, *τ_rθ_* = 0	10	0	*u* ≠ 0, *τ_rθ_* = 0	10	0
	elastic 2 (E^2^)	*v* ≠ 0, *σ_r_* = 0	0	10^2^	*v* ≠ 0, *σ_θ_* = 0	0	10^2^
	elastic 3 (E^3^)	*u* ≠ 0, *v* ≠ 0	10^3^	10^3^	*u* ≠ 0, *v* ≠ 0	10^3^	10^3^

### 3.1. In-plane vibration of rectangular plates

In the following calculations, all the terms of the Fourier series for the displacement fields are truncated into *M* = *N* = 10. As far as the accuracy of the present method is concerned, the converged solutions of the present method are in excellent agreement with both the reference data and the finite-element results. Table 4 gives the normalized frequency parameter *Ω* = *ωb*(*ρ/G_xy_*)^1/2^/*π* for rectangular plates under different boundary conditions (*E_x_/E_y_* = 2), which agree very well with data from the literature. Table 5 considers the influence of different values of *E_x_/E_y_* for a S^2^CS^2^F orthotropic rectangular plate. The data obtained in [6,11] are also provided for comparison. All the results are in good agreement with data in previous studies.

**Table 4.** Normalized frequency parameter for square plates with various boundary conditions (*E_x_/E_y_* = 2).

	mode no.								
B. C.	1	2	3	4	5	6	7	8	9
S^2^S^1^S^2^C	0.7070	1.1617	1.9170	2.0777	2.1209	2.3778	2.6047	2.8188	3.050
	*0.7071^a^*	*1.1619*	*1.9177*	*2.0779*	*2.1213*	*2.3789*	*2.6052*	*2.8200*	*3.0508*
S^2^S^2^S^2^C	1.4141	1.5223	2.0081	2.2931	2.3491	2.8060	2.8281	2.9749	3.1979
	*1.4142^b^*	*1.5228*	*2.0815*	*2.2932*	*2.3493*	*2.8060*	*2.8284*	*2.9750*	*3.1975*
S^2^CS^2^C	1.4142	1.6169	2.1208	2.3235	2.7838	2.8272	2.8513	3.0447	3.2114
	*1.4141^a^*	*1.6173*	*2.1222*	*2.3238*	*2.7853*	*2.8284*	*2.8522*	*3.0457*	*3.2116*
S^2^S^1^S^2^F	0.8956	1.4142	1.4322	1.8517	2.0708	2.1814	2.3409	2.7901	2.8151
	*0.8957^a^*	*1.4142*	*1.4324*	*1.8517*	*2.0720*	*2.1818*	*2.3417*	*2.7893*	*2.8158*
S^2^CS^2^F	0.7070	1.0299	1.8749	1.8759	2.1209	2.2721	2.4267	2.5573	2.7935
	*0.7071^a^*	*1.0301*	*1.8746*	*1.8768*	*2.1213*	*2.2731*	*2.4279*	*2.5577*	*2.7932*
S^2^S^1^S^1^C	0.8085	1.3921	1.6055	2.0015	2.2484	2.3801	2.5611	3.0011	3.0212
	*0.8087^b^*	*1.3928*	*1.6058*	*2.0019*	*2.2488*	*2.3809*	*2.5615*	*3.0016*	*3.0219*
S^2^S^2^S^1^C	1.0607	1.4256	1.8428	1.8806	2.939	2.6480	2.7361	2.9937	3.0715
	*1.0611* *^b^*	*1.4261*	*1.8433*	*1.8811*	*2.5402*	*2.6482*	*2.7364*	*2.9942*	*3.0720*
S^2^CS^1^C	1.3859	1.4422	1.9311	2.2449	2.7033	2.7563	2.7639	3.2421	3.3282
	*1.3864^b^*	*1.4426*	*1.9315*	*2.2452*	*2.7036*	*2.7566*	*2.7648*	*3.2425*	*3.3286*
S^2^S^1^S^1^F	0.4029	1.0563	1.3770	1.3972	1.7724	1.8627	2.3221	2.5317	2.5750
	*0.4029* *^b^*	*1.0566*	*1.3771*	*1.3974*	*1.7727*	*1.8629*	*2.3219*	*2.5320*	*2.5753*
S^2^S^2^S^1^F	0.6981	1.0070	1.4124	1.4232	1.9817	2.1600	2.3307	2.3924	2.8964
	*0.6983* *^b^*	*1.0073*	*1.4126*	*1.4234*	*1.9821*	*2.1603*	*2.3303*	*2.3927*	*2.8968*

^a^Results in italic font from [11].

^b^Results in italic font from [6].

**Table 5.** Normalized frequency parameter for orthotropic square plates of different stiffness ratios *E_x_/E_y_* under S^2^CS^2^F boundary condition.

	mode no.									
*E_x_*/*E_y_*	1	2	3	4	5	6	7	8	9	10
3	0.7070	1.0717	1.9367	2.0823	2.1210	2.5607	2.6650	2.7598	2.8758	3.4396
	*0.7071^a^*	*1.0719*	*1.9364*	*2.0827*	*2.1213*	*2.5624*	*2.6651*	*2.7612*	*2.8726*	*3.4389*
6	0.7071	1.1243	2.0061	2.1212	2.2154	2.7743	2.9626	3.3606	3.5072	3.5353
	*0.7071^b^*	*1.1243*	*2.0044*	*2.1213*	*2.2156*	*2.7737*	*2.9541*	*3.3614*	*3.5099*	*3.5355*
9	0.7071	1.1461	2.0336	2.1212	2.2532	2.8129	2.9956	3.5101	3.5354	3.5760
	*0.7071^a^*	*1.1460*	*2.0310*	*2.1213*	*2.2533*	*2.8119*	*2.9866*	*3.5103*	*3.5355*	*3.5717*

^a^Results in italic font from [11].

^b^Results in italic font from [6].

### 3.2. Annular sectorial plates

In this section, the convergence of the method is studied first, followed by some benchmark examples that demonstrate the excellent accuracy and reliability of the current approach, and then some computational examples using the generalized model are presented. Unless otherwise stated, the values of the following variables are used: *R*_1_ = 1 m, *ϕ* = 90*°*, and *h*/*R*_1_ = 0.001.

In the convergence study, choosing an appropriate number of terms in the truncated series is important. Table 6 shows the first four non-dimensional frequency parameters of annular sectorial plates with free boundary condition at all the edges for different number of terms. The inner--outer radius ratio is *R*_0_/*R*_1_ = 1/2. Table 6 shows the trend of the frequency parameters with increasing number of terms in the truncated series. 10 × 10 terms in the truncated series give satisfactory accuracy as will be shown in the numerical examples in tables 7 and 8.

**Table 6.** Normalized frequency parameters for annular plates with complete free boundary conditions.

	mode no.					
M × N	1	2	3	4	5	6
7 × 7	1.0316	1.7348	2.0502	3.0598	3.1928	3.4189
8 × 8	1.0303	1.7348	2.0502	3.0597	3.1857	3.4101
9 × 9	1.0303	1.7348	2.0497	3.0597	3.1827	3.4096
10 × 10	1.0302	1.7347	2.0485	3.0597	3.1820	3.4086
11 × 11	1.0302	1.7347	2.0485	3.0597	3.1820	3.4086

**Table 7.** Frequency parameters for annular sectorial plate with various classical boundary condition. Note: FE_AB_ represents results obtained from ABAQUS; FE_AN_ represents results from ANASYS.

		mode no.							
B.C.	method	1	2	3	4	5	6	7	8
CCCC	present	3.3677	4.4791	5.8203	5.9742	6.7084	7.2168	7.7834	8.6131
	*FE_AB_* [25]	*3.3692*	*4.4807*	*5.8240*	*5.9810*	*6.7152*	*7.2240*	*7.7894*	*8.626*
S^1^S^1^S^1^S^1^	present	1.5882	3.063	3.6308	4.4535	5.8097	6.0961	4.9339	6.4738
	*FE_AN_* [25]	*1.5884*	*3.0641*	*3.6320*	*4.4572*	*4.9362*	*5.8163*	*6.1036*	*6.4793*
S^2^S^2^S^2^S^2^	present	1.3469	2.3639	2.8909	3.4244	3.5297	4.5766	4.7263	5.7089
	*FE_AB_* [25]	*1.347*	*2.3646*	*2.8920*	*3.4255*	*3.5307*	*4.5787*	*4.7307*	*5.7165*
FFFF	present	1.0311	1.7367	2.0504	3.0618	3.1817	3.4099	4.303	4.5766
	*FE_AN_* [25]	*1.0312*	*1.7370*	*2.0513*	*3.0630*	*3.1835*	*3.4108*	*4.3054*	*4.5788*

**Table 8.** Normalized frequency parameters for rectangular and annular sectorial plates by using the generalized model (*b* = *φ* = *n*/2, Material D).

		mode no.						
shape	B.C.	method	1	2	3	4	5	6
rectangular	CCCC	present	1.5612	2.1871	2.7527	3.0299	2.1871	3.7806
	CFCF	present	1.2351	1.4659	1.9496	2.061	1.4659	2.9473
	FFFF	present	0.9453	1.5087	1.7516	2.2831	1.5087	2.4401
	CCCF	present	1.3177	1.7386	2.2115	2.5773	3.0693	3.4266
	E^1^E^1^E^1^E^1^	present	1.5532	1.7584	2.1172	2.2024	2.7417	2.9948
	E^2^E^2^E^2^E^2^	present	0.9884	1.9771	2.0642	2.4233	2.5015	2.9662
	E^3^E^3^E^3^E^3^	present	2.6364	2.6921	3.0323	3.6336	3.6488	4.3635
annular sectorial	CCCC	present	3.1711	4.2193	4.5756	4.6018	5.0437	5.4068
		[25]	*3.1706*	*4.2189*	*4.5759*	*4.5995*	*5.0436*	*5.4038*
	CFCF	present	2.5339	2.9852	3.9964	4.3373	4.3721	4.5228
		[24]	*2.5249*	*2.9815*	*4.0516*	*4.3641*	*4.4014*	*4.5238*
	FFFF	present	1.0433	1.7875	1.9884	2.9706	2.9783	3.1146
		[28]	*1.0433*	*1.7875*	*1.9884*	*2.9706*	*2.9782*	*3.1144*
	CCCF	present	2.6831	3.6011	4.3020	4.4187	4.6583	4.9090
		[25]	*2.6788*	*3.6030*	*4.3005*	*4.4002*	*4.6596*	*4.9081*
	E^1^E^1^E^1^E^1^	present	1.6800	2.5966	2.7452	2.9678	3.2766	3.6939
	E^2^E^2^E^2^E^2^	present	0.9181	1.2303	1.994	2.8782	3.0225	3.7713
	E^3^E^3^E^3^E^3^	present	3.1658	4.2083	4.5641	4.5877	5.0338	5.3970

In table 7, the results of an annular plate with different classical boundary conditions are compared to data from the literature. The results by ABAQUS-V6.12 and ANASYS-V14.5 are from [25]. Table 7 shows excellent agreement between the current model and existing data. These agreements prove that the present method with the adoption of the logarithmic radial variable is accurate and efficient in solving the in-plane vibration problems for annular sectorial plates. The presented method improves the efficiency for the sectorial plate in two aspects. First, the stiffness matrix and the mass matrix in equation (2.34) has the explicit form, which does not need the tedious numerical integration process [25], so it is helpful in generating the global matrices. Second, in equation (2.25), the terms in the truncated series of this paper are *M* = *N* = 10, while the chosen number of *M* and *N* are 15 in [25]. The present method significantly reduces the computation cost of the eigen-problems for the matrix dimension in equation (2.34) is 4/9 of that in [25], which does not use the logarithmic radial variable.

Most existing techniques can only handle one kind of the boundary condition and geometrical shape, but the current method can easily accommodate changes in geometry and boundary conditions.

### 3.3. In-plane vibration of rectangular and annular sectorial plates problems solved by the generalized model

In this section, the generalized model is used in several numerical examples. The parameters *ξ*_max_, *η*_max_ are assigned the same values for plates of different shapes. For example, when the aspect ratio of the outer--inner radius for the annular sectorial plate is *R*_1_/*R*_0_ = 2, the length ratio of the rectangular plate is *b/a* = ln(2). In the *η* direction, the width of the rectangular plate is set to be equal to the angle of the sectorial plate, e.g. *b = ϕ* = π/2. The calculated frequency parameters for the two shapes are listed in table 8. It is shown from table 8 that the generalized model accommodates both the rectangular and annular sectorial shapes, and the results agree very well with data from the literature [24,25,28].

## 4. Concluding remarks

(1) A variable transformation by adopting the logarithmic radial variable significantly simplifies the basic theory for in-plane vibration of annular sectorial plate. This simplification makes it possible to formulate the basic theories for annular sectorial and rectangular plates in a uniform framework.
(2) In the generalized model, the improved Fourier–Ritz expansion of the displacements are expressed by admissible trigonometric functions. Due to the orthogonality of the modified Fourier series, the global stiffness and mass matrices can be obtained explicitly by using the integration formulae in the electronic supplementary material.
(3) The appropriate spring stiffness values for various boundary conditions for rectangular and annular sectorial plates are discussed and provided in table 3.
(4) The number of terms in the truncated Fourier series for displacement fields are 10 × 10 for both shapes, and the numerical results show that the error of the present generalized model is universally less than 0.5%.

## Supplementary Material

Supplementary Material

## Data accessibility

Supporting data in this work can be accessed via the following links: http://dx.doi.org/10.5061/dryad.hd7tm [33], http://qishigreat.blog.hexun.com/111979175_d.html.
