# Supplementary material for: A generalized solution procedure for in-plane free vibration of rectangular plates and annular sectorial plates
Source: R Soc Open Sci. 2017 Aug 16;4(8):170484. doi: 10.1098/rsos.170484 (PMC5579111; doi:10.1098/rsos.170484)
Supplement: Supplementary Material [file rsos170484supp1.doc]

**Elementary Supplement Materials**

Useful integration

$\int_{0}^{L} \cos\left(\lambda_{m}x\right)\cos(\lambda_{n}x)\text{d}x=\left\{\begin{matrix} 0\text{ }m\neq n \\ \frac{L}{2}\text{ }m=n\neq0 \\ L\text{ }m=n=0 \end{matrix}\right.$ （ A1）

$\int_{0}^{L} \sin\left(\lambda_{m}x\right)\sin(\lambda_{n}x)\text{d}x=\left\{\begin{matrix} 0\text{ }m\neq n\text{ } \\ \frac{L}{2}\text{ }m=n\neq0 \\ 0\text{ }m=n\text{=0} \end{matrix}\right.$ （ A2）

$\int_{0}^{L} \sin\left(\lambda_{m}x\right)\cos(\lambda_{n}x)\text{d}x=\left\{\begin{matrix} \frac{(1-(-1)^{m+n})Lm}{(m^{2}-n^{2})\pi}\text{ }m\neq n \\ 0\text{ }m=n \end{matrix}\right.$ （ A3）

$$\begin{matrix} \int_{0}^{L} \cos\left(\lambda_{m}x\right)\cos(\lambda_{n}x)\text{e}^{ax}\text{d}x=[(-1)^{m+n}\text{e}^{aL}-1]\frac{aL^{2}}{2}\left(\frac{1}{a^{2}L^{2}+(m+n)^{2}\pi^{2}}+\frac{1}{a^{2}L^{2}+(m-n)^{2}\pi^{2}}\right)\text{ } \\ \int_{0}^{L} \sin\left(\lambda_{m}x\right)\sin(\lambda_{n}x)\text{e}^{ax}\text{d}x=[(-1)^{m+n}\text{e}^{aL}-1]\frac{aL^{2}}{2}\left(\frac{1}{a^{2}L^{2}+\pi^{2}(m-n)^{2}}-\frac{1}{a^{2}L^{2}+\pi^{2}(m+n)^{2}}\right)\text{ } \\ \int_{0}^{L} \sin\left(\lambda_{m}x\right)\cos(\lambda_{n}x)\text{e}^{ax}\text{d}x=\left(1-(-1)^{m+n}\text{e}^{aL}\right)m\pi L\frac{a^{2}L^{2}+m^{2}\pi^{2}-n^{2}\pi^{2}}{\left(a^{2}L^{2}+(m-n)^{2}\pi^{2}\right)\left(a^{2}L^{2}+(m+n)^{2}\pi^{2}\right)}\text{ } \end{matrix}$$

(*a*=1, 2) （ A4）

where $\lambda_{m}=\frac{m\pi}{L},\lambda_{n}=\frac{n\pi}{L}$.
